# Comprehensive genomics analysis of aging related gene signature to predict the prognosis and drug resistance of colon adenocarcinoma

**DOI:** 10.3389/fphar.2023.1121634

**Published:** 2023-02-28

**Authors:** Jubin Feng, Fengyihuan Fu, Yuqiang Nie

**Affiliations:** ^1^ The First Affiliated Hospital, Jinan University, Guangzhou, China; ^2^ Department of Gastroenterology, The Second Affiliated Hospital of Guangzhou Medical University, Guangzhou, China; ^3^ The Second Affiliated Hospital of Guangzhou Medical University, Guangzhou, China; ^4^ Department of Gastroenterology, School of Medicine, Guangzhou First People’s Hospital, South China University of Technology, Guangzhou, China

**Keywords:** colon adenocarcinoma, senescence, subtypes, prognosis, risk score, nomogram, chemotherapy drugs

## Abstract

**Background:** Colon adenocarcinoma (COAD) is a heterogeneous tumor and senescence is crucial in the occurrence of cancer. This study aimed to identify senescence-based subtypes and construct a prognostic signature to predict the prognosis and guide immunotherapy or chemotherapy decisions for COAD patients.

**Methods:** Based on the single-cell RNA sequencing (scRNA-seq) data of 13 samples from the Gene Expression Omnibus (GEO) database, we assessed cellular senescence characteristics. Transcriptome data, copy number variations (CNVs) and single nucleotide variations (SNVs) data were obtained from The Cancer Genome Atlas (TCGA) database. GSE39582 and GSE17537 were used for validation. Senescence subtypes were identified using unsupervised consensus clustering analysis, and a prognostic signature was developed using univariate Cox analysis and least absolute shrinkage and selection operator (LASSO). Response of risk groups to chemotherapy was predicted using the half-maximal inhibitory concentration (IC50) values. We further analyzed the relationship between risk gene expression and methylation level. The prediction performance was assessed by nomogram.

**Results:** Senescence-related pathways were highly enriched in malignant cells and bulk RNA-seq verified cellular senescence. Three senescence subtypes were identified, in which patients in clust3 had poorest prognosis and higher T stage, accompanied with higher tumor mutation burden (TMB) and mutations, activated inflammatory response, more immune cell infiltration, and higher immune escape tendency. A senescence-based signature using 11 genes (MFNG, GPRC5B, TNNT1, CCL22, NOXA1, PABPC1L, PCOLCE2, MID2, CPA3, HSPA1A, and CALB1) was established, and accurately predicted a lower prognosis in high risk patients. Its robustness was validated by external cohort. Low risk patients were more sensitive to small molecule drugs including Erlotinib, Sunitinib, MG-132, CGP-082996, AZ628, Sorafenib, VX-680, and Z-LLNle-CHO. Risk score was an independent prognostic factor and nomogram confirmed its reliability. Four risk genes (CALB1, CPA3, NOXA1, and TNNT1) had significant positive correlation with their methylation level, while six genes (CCL22, GPRC5B, HSPA1A, MFNG, PABPC1L, and PCOLCE2) were negatively correlated with their methylation level.

**Conclusion:** This study provides novel understanding of heterogeneity in COAD from the perspective of senescence, and develops signatures for prognosis prediction in COAD.

## Introduction

Colorectal cancer (CRC), is the most common diagnosed gastrointestinal malignant tumor, and ranks third in the morbidity and second in the mortality with an estimated 3.2 million new COAD cases in 2040 worldwide (Xi and Xu, 2021). The prevalence of CRC is 0.56 million in 2020, and will increase to 0.91 million in 2040 in China (Xi and Xu, 2021). Among these, colon adenocarcinoma (COAD) accounts for 90% of cases (Munro et al., 2018). Patients with Stage 1-2 have a 5-year survival rate of 82%–94%, while it reduces to 67% for patients with stage 3 and advanced metastatic or stage 4 have a dismal 5-year survival rate of only 11% (Sagaert et al., 2018). Various treatments such as radical surgery followed by adjuvant chemotherapies can be used for treatment of resectable COAD patients, and palliative chemo- or radiotherapy is optimal for unresectable COAD patients to prolong their life. It has been recognized that COAD is a malignancy with intertumor and intratumor heterogeneity, which contribute to difference of prognosis and therapy response (Punt et al., 2017). Hence, it is great of importance to stratify patients with COAD and develop novel markers to accurately predict prognosis and therapy response.

Over the past decades, high throughput sequencing technology has been widely used in various fields of biology and medicine, greatly promoting relevant research and clinical application (Lightbody et al., 2019). The traditional RNA sequencing technology (bulk RNA-seq) is applied to determine gene expression profiles, isoform expression, alternative splicing and single-nucleotide polymorphisms on basis of tissue samples, which contains various cell types (Kuksin et al., 2021). On the contrast, single-cell RNA sequencing (scRNA-seq), a novel technology can detect the gene expression patterns for each transcript within single cell and distinguish cell subtypes (Lähnemann et al., 2020). Recently, scRNA-seq has been employed widely used in different cell type of various species, especially in human and mouse, to assess biological variability (Papalexi and Satija, 2018).

Cellular senescence is a cell state of cell cycle arrest that can eliminate damaged cells and promote tissue remodeling. Cellular senescence is predominantly elicited in response to intrinsic and extrinsic stimulus, such as oncogene activation, stress, DNA damage, CDKN2A locus derepression, mitochondrial dysfunction (Hernandez-Segura et al., 2018). Unfortunately, compelling evidence has suggested that cellular senescence is implicated in pathological status, in which senescence-associated secretory phenotype (SASP) affects the clearance of senescent cells and further results the decline of tissue function (Muñoz-Espín and Serrano, 2014), and secret pro-inflammatory cytokines including interleukin (IL)-6 and IL-8, chemokines and growth factors, which contributes to tumorigenesis in aged organisms (Herranz and Gil, 2018). Cellular senescence has been studied in various cancer types and compelling evidences have revealed that cellular senescence is associated with cancer prognosis (Dai et al., 2022a; Domen et al., 2022a; Domen et al., 2022b). Development of senescence-related classification and characterization of senescence-based signature have attracted much attention in tumor research (Feng et al., 2022a; Hong et al., 2022). However, the mechanisms of cellular senescence in COAD, as well as the specific prognostic signatures are poorly understood. Therefore, this study identified senescence-based subtypes based on scRNA-seq and shed novel insights into potential roles of cellular senescence in COAD heterogeneity. We further constructed a prognostic risk model in The Cancer Genome Atlas (TCGA)-COAD, which offered a novel approach to predict clinical outcomes in patients with COAD.

## Material and methods

### Single-cell RNA sequencing (scRNA-seq) data collection and pre-processing

The scRNA-seq expression profiles of 13 samples (GSE161277) (Zheng et al., 2022) were downloaded from Gene-Expression Omnibus (GEO; https://www.ncbi.nlm.nih.gov/geo/) database. To comprehensively understand the profile of cellular senescence-related genes in COAD patients, we filtered scRNA-seq data by setting each gene expressed in at least three cells, and each cell expressing at least 250 genes. The percentage of mitochondria and rRNA in each cell was calculated using the PercentageFeatureSet function ensuring 100 < genes < 6,000 and mitochondrial content <5% in each cell. Data of 13 samples were normalized using log-normalization method, and the FindVariableFeatures function was used to identify variable features based on variance stabilization transformation (“vst”) and select highly variable genes.

### Transcriptome data collection and pre-processing

The gene expression profiles and clinical information of COAD were obtained from The Cancer Genome Atlas (TCGA) database (https://portal.gdc.cancer.gov/) project, including 432 tumor samples and 41 para-carcinoma tissue samples. The RNA-seq data standardization method was TPM normalization. To process TCGA-COAD data, samples lacking clinical follow-up information, survival time, and survival status were eliminated from further analysis, and all samples with survival time more than 0 days. Ensembl gene IDs were further transformed into gene symbol IDs. Then, the gene with multiple gene symbol IDs was normalized as median. We also downloaded the gene expression profiles of 573 COAD samples in GSE39582 (Marisa et al., 2013) and 55 COAD samples in GSE17537 (Xiao et al., 2022) from GEO database. Among these, clinical follow-up information, survival time, and survival status were excluded from this study. We converted ensembl gene IDs to gene symbol IDs. The probe related to several genes was removed, and the gene with multiple probes was expressed as median.

Masked copy number segment data of COAD were collected from TCGA and progressed by gistic2 software. Single nucleotide variations (SNVs) data of COAD that was derived using mutect2 software were obtained from TCGA cohort. Moreover, we obtained methylation data from TCGA. Methylation data was processed with following steps: 1) KNN function in “impute” R package was used to complete the NA value. 2) We converted beta value to M value. 3) We removed the cross-reactive CpG sites as previously reported (Chen et al., 2013). 4) We removed the unstable genomic methylation sites, that was, removed the CpG sites and single nucleotide sites on the sex chromosome. 5) Tumor samples (solid tumors) were retained in this study.

### Collection of senescence-related pathways

Senescence-related pathways were retrieved in the Molecular Signatures Database (MSigDB, https://www.gsea-msigdb.org/gsea/index. jsp).

## Screening for cell subpopulations and marker genes

Subsequently, all genes were scaled through the ScaleData function, and principal components analysis (PCA) was conducted to reduce the dimensionality. The FindNeighbors and FindClusters functions were used to cluster cells (Resolution = 0.1). Further, we reduced the t-distributed stochastic neighbor embedding (TSNE) dimensionality using RunTSNE founction and then annotated the cell subpopulations with some classic markers of immune cells (Zheng et al., 2022). The FindAllMarkers function was employed to identify marker genes with logFC = 0.5 and Minpct = 0.5 under the statistical threshold of adjusted *p* < 0.05. “clusterProfiler” package (Yu et al., 2012) was implemented for Kyoto Encyclopedia of Genes and Genomes (KEGG) enrichment analysis.

### Cellular senescence characteristics in tumor microenvironment (TME) of single cell

The number of DNA copies was calculated by “copycat” package (Gao et al., 2021) under the threshold of at least 5 genes in each chromosome. We distinguished aneuploidy (malignant cells) and diploid (non-malignant cells) with at least 25 genes selected for each segment and KS. cut = 0.15. We downloaded the cellular senescence-related pathways from gene set enrichment analysis (GSEA, http://www.gsea-msigdb.org/gsea/index.jsp), and calculated single sample GSEA (ssGSEA) scores of aneuploidy and diploid through “GSVA” package (Hänzelmann et al., 2013). The distribution was compared using the wilcox. test, and *p* < 0.05 was considered statistically significant.

### Verification of cellular senescence based on bulk RNA-seq data

Furthermore, we used bulk RNA-seq data to analyze abnormal cellular senescence in tumor and normal COAD samples. GSEA was applied to performed pathway enrichment analysis, and ssGSEA scores of cellular senescence-related pathways were calculated in tumor and normal COAD samples. The distribution was compared using the wilcox. test.

### Identification of senescence subtypes

Based on the above analysis, genes in GOBP_REPLICATIVE_SENESCENCE, REACTOME_CELLULAR_SENESCENCE, REACT OME_DNA_DAMAGE_TELOMERE_STRESS_INDUCED_SENESC ENCE, and KEGG_P53_SIGNALING_PATHWAY were selected for univariate Cox regression analysis using “survival” package (Therneau and Lumley, 2015) in R. Candidates with *p* < 0.05 were considered as prognosis-related genes. A consensus clustering analysis was performed to categorize the 432 TCGA-COAD samples based on the expression profiles of the 16 senescence-related genes using “Consensus ClusterPlus” package (Wilkerson et al., 2013) with “Partitioning Around Medoids” (PAM) algorithm (Kaufman and Rousseeuw, 1990) and Euclidean distancing, in procedures with 500 bootstraps containing 80% COAD patients. 2–10 clusters were tested. The cumulative distribution function (CDF) and consensus matrix were performed identify the optimal subtypes. Kaplan-Meier curves of identified subtypes were generated in TCGA cohort and GSE39582 cohort.

### Analysis of clinicopathologic characteristics among senescence subtypes

We further compared the distributions of clinicopathologic characteristics (gender, T stage, N stage, M stage, Stage, age, and survival status) among three senescence subtypes in TCGA cohort using Chi square test. Besides, the distributions of subtypes in T stage and survival status (alive or dead) were also analyzed using Sankey diagram.

### Differences in mutation characteristics among senescence subtypes

We integrated copy number variations (CNVs) of TCGA-COAD patients through gistic2 software with a confidence level of 0.9 and hg38 as the reference genome to analyze the differences of CNVs among the three subtypes. “maftools” package (Mayakonda et al., 2018) was employed to analyze SNVs data in TCGA cohort. Additionally, comparisons of TMB and the number of genetic mutations were carried out using wilcox. test among three subtypes.

### Relationship between senescence subtypes and enriched pathway characteristics

To evaluate the relationship between senescence subtypes and epithelial-to-mesenchymal transition (EMT), we calculated ssGSEA scores of EMT in each TCGA-COAD sample on basis of 200 genes of HALLMARK_EPITHELIAL_MESENCHYMAL_TRANSITION in MSigDB (Yu et al., 2021). We calculated hypoxia score of genes of HALLMARK_HYPOXIA using ssGSEA method. Based on 24 genes as previously reported (Masiero et al., 2013), we scored angiogenesis by ssGSEA method. Differential analysis of these ssGSEA scores were performed using wilcox. test. Meanwhile, 10 tumor-related pathways were obtained (Sanchez-Vega et al., 2018) and the enrichment score was calculated by ssGSEA, followed by kruskal. test for comparisons.

### Relationship between senescence subtypes and immune characteristics

We evaluated the immune cell infiltration in TCGA cohort by ESTIMATE algorithm, and calculated the score of 28 kinds of immune cells (Charoentong et al., 2017) by ssGSEA. Afterwards, we downloaded the genes related to inflammation through GSEA and calculated their ssGSEA scores. Comparisons were analyzed using kruskal. test. The tumor immune dysfunction and exclusion (TIDE) is a computational method that can determine the signatures of T cell dysfunction by using gene expression profiling in tumors interacts with the cytotoxic T lymphocytes infiltration level to affect patient survival and response to immunotherapy (Jiang et al., 2018). A high TIDE score indicates a low response rate to immune checkpoint inhibition (ICI) therapy. Thus, the TIDE algorithm (http://tide.dfci.harvard.edu/) was employed to predict the potential clinical effects of immunotherapy in subtypes.

### Construction and validation of senescence-based risk model

To identify the differential expressed genes (DEGs), “limma” package (Ritchie et al., 2015) in R was applied to perform differential analysis when clust1 vs. non-clust1, clust2 vs. non-clust2 and clust3 vs. non-clust3. Under the threshold of *p* < 0.05 and |log2 (Fold Chage)| > log2 (1.5), 2,085 DEGs were identified and selected for univariate Cox regression analysis using coxph function embedded in “survival” package, and candidates with *p* < 0.005 were selected as genes that have greater impact on prognosis. To reduce the number of genes, the LASSO Cox regression was performed using “glmnet” package (Hastie et al., 2021) in R. Stepwise multivariate regression analysis with stepwise Akaike information criterion (AIC) was used to determine genes for risk model construction.

The risk score formula related to the prognostic signature was as follows: RiskScore = 0.417*MFNG + 0.424*GPRC5B + 0.137*TNNT1−0.389*CCL22 + 0.308*NOXA1 + 0.149*PABPC1L + 0.338*PCOLCE2 + 0.337*MID2−0.215*CPA3 + 0.261*HSPA1A + 0.161*CALB1. After calculating risk score in TCGA cohort, “timeROC” package (Blanche, 2015) was employed to carry out receiver operating characteristic (ROC) analysis with areas under the ROC curve (AUCs) for 1, 3, and 5 years. Finally, risk score was standardized as zscore, and TCGA-COAD samples were divided into high-risk group (zscore >0) and low-risk group (zscore <0). Kaplan-Meier curves were generated between high- and low-risk groups.

Associations of senescence-based risk score with clinicopathologic characteristics and biological characteristics.

To explore the relationship between RiskScore score and clinical characteristics of COAD patients, we analyzed the differences of risk score among clinicopathologic characteristics including gender, age, T stage, N stage, M stage, Stage, and clusters in TCGA-COAD cohort. Additionally, we performed correlation analysis between senescence-based risk score and biological characteristics (hypoxia, angiogenesis, and metastasis) with rcorr function in “Hmisc” package (Harrell and Harrell, 2019). Further, we used “GSVA” package to score pathways in KEGG, and performed correlation analysis between senescence-based risk score and pathways with |cor| > 0.2 and *p* < 0.05. We compared the scores of senescence-related pathways between high- and low risk groups. Wilcox. test was applied for comparisons.

## Prediction of responsiveness to chemotherapy

To predict the responsiveness to traditional chemotherapy drugs, the half-maximal inhibitory concentration (IC50) values were evaluated using the “pRRophetic” package. Comparisons of IC50 values between high- and low-risk groups were performed using wilcox. tests.

### Relationship between risk gene expression and methylation

Based on the methylation data of the TCGA dataset, we constructed the methylation level of the CpG sites in the risk model and calculated the mean values of methylation level at different CpG sites of the same gene. The relationship between risk gene expression and the methylation level was analyzed using Pearson correlation analysis.

### Construction of nomogram

Furthermore, the univariate and multivariate Cox regression analysis were utilized to determine whether senescence-based risk score is an independent predictor of prognosis. To predict the clinical outcomes of COAD patients, a nomogram based on risk score and clinicopathological characteristics was constructed with calibration curve. To evaluate the accuracy and reliability of this model, decision curve analysis (DCA) was established.

### Statistical analysis

Data was processed and analyzed using (version 3.6.0, https://www.r-project.org/) and Seurat R package (Gribov et al., 2010) (version 3.6.3, https://satijalab.org/seurat/). Wilcox. test or kruskal. test was applied to determine the significant differences and *p* < 0.05 was considered statistically significant. Log-rank test was used to determine the statistically significant for Kaplan-Meier curves.

## Results

### Single cell RNA-seq analysis and marker gene recognition of COAD

Supplementary Figure S1A showed the cell number of 13 samples before and after filtering. As displayed in Supplementary Figure S1B, 13 samples overlapped significantly between the TSNE diagrams. After PCA for dimension reduction (Supplementary Figure S1C, D), we select dim = 35 for further analysis.

**FIGURE 1:**
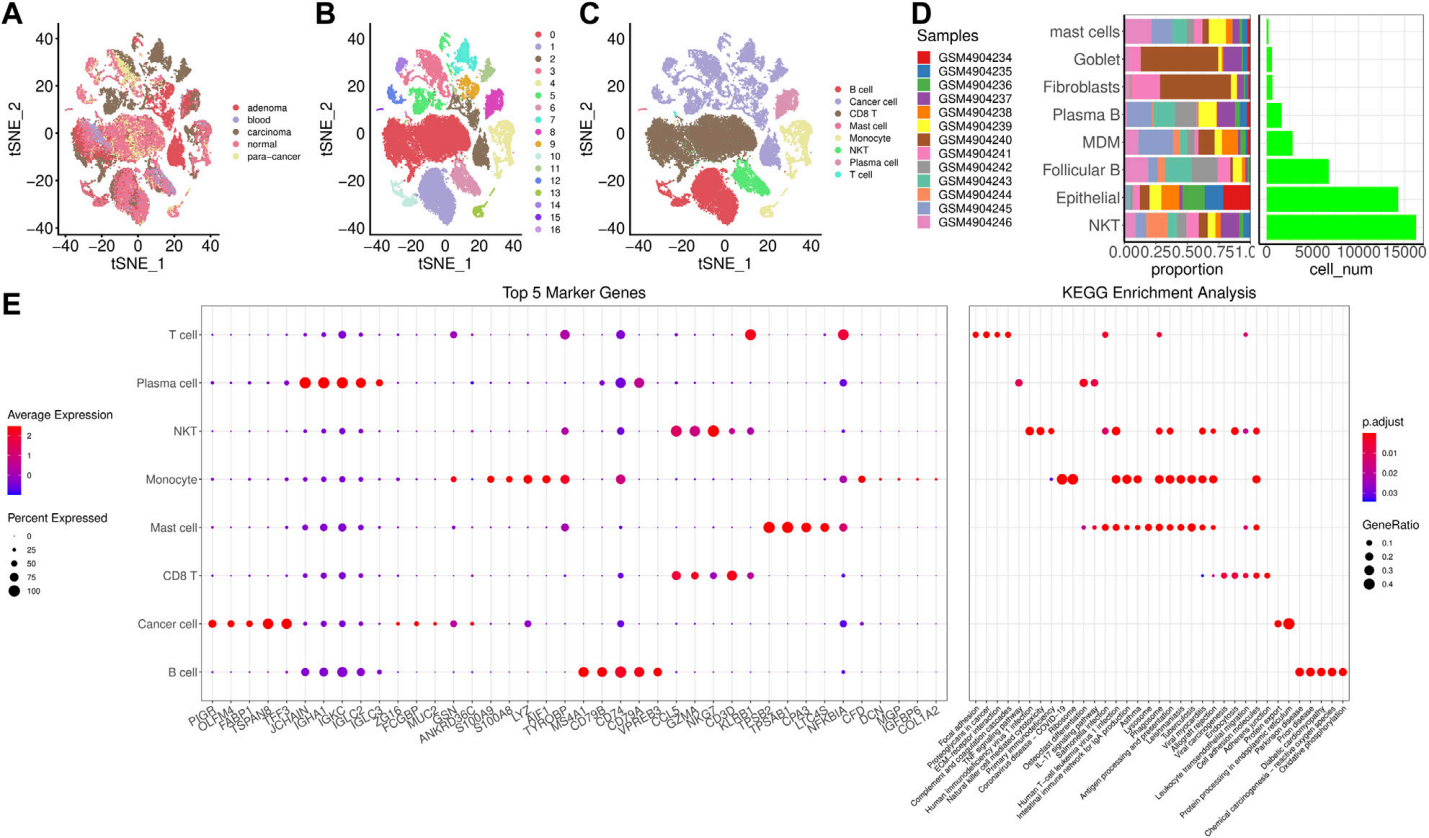
Screening for cell subpopulations and marker genes. **(A)**, TSNE diagrams of 5 samples. **(B)**, TSNE diagrams of 17 cell subpopulations. **(C)**, TSNE diagrams of 8 subpopulations after annotation. **(D)**, The number of cells of each subpopulation and its proportion in different samples. **(E)**, Top 5 maker genes in each subpopulation and the enrichment analysis.

We clustered cells based on dim = 35 and obtained 17 cell subpopulations. Figure 1A showed t-SNE-maps of adenoma, blood, carcinoma, normal and para-cancer samples. Figure 1B portrayed 17 cell subpopulations after clustering. Then, we annotated the cell subpopulations with some classic markers of immune cells. Supplementary Figure S2 provided TSNE diagram of marker gene expression. Figure 1C showed the clustering characteristics of annotated cell subpopulations. Subpopulations 2, 3, 5, 7, 9, 11, and 12 were epithelial cells expressing EPCAM; Subpopulations 0 and 6 were natural killer (NK) T cells expressing CD3D, KLRD1, and CD8A. Subpopulations 1 and 10 were follicular B cells expressing MS4A1; Subpopulation 8 was plasma B cells expressing MZB1; Subpopulation 4 was monocyte derived macrophages (MDMD) expressing CD68, CD14, and FCGR3A; Subpopulation 13 was fibroblasts expressing DCN; Subpopulations 15 and 16 were mast cells expressing KIT. Accordingly, we counted the number of cells of each subpopulation and calculated its proportion in different samples (Figure 1D). The subpopulations epithelial and NK T had larger number of cells than others. Figure 1E showed top 5 maker genes in each subpopulation and the enrichment analysis showed that marker genes were closely associated with human T-cell leukemia virus 1 infection, Th17 cell differentiation, hematopoietic cell lineage, and Th1 and Th2 cell differentiation.

### Cellular senescence characteristics in TME

**FIGURE 2:**
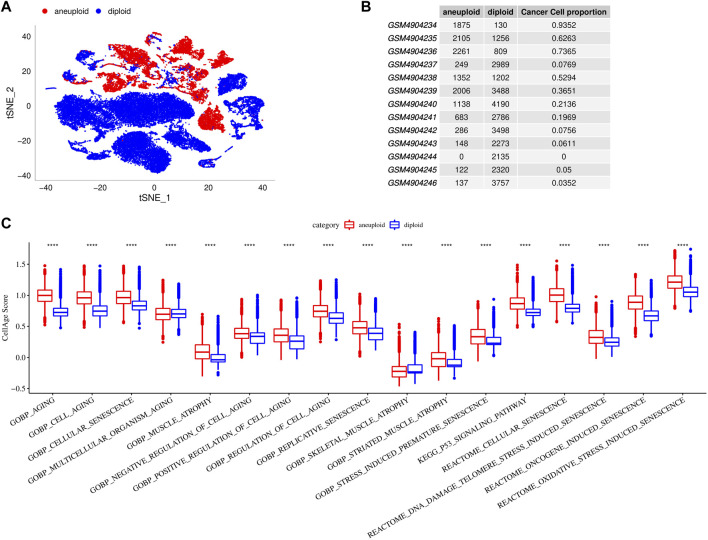
Cellular senescence characteristics in single cell TME. **(A)**, TSNE diagrams of malignant cells and non-malignant cells in single cell. **(B)**, Cell proportions of malignant cells and non-malignant cells in 13 samples. **(C)**, Comparisons of senescence-related pathway scores between malignant cells and non-malignant cells.

To characterize cellular senescence in TME of single cell, we distinguished aneuploidy and diploid in cell subpopulations. The results revealed that there were 12,362 aneuploid (malignant cells) and 30,833 diploid (non-malignant cells), and their TNSE-maps were shown in Figures 2A, B suggested that there are more malignant cells in cancer tissues, but fewer malignant cells in para-cancer tissues. Further we calculated the cellular senescence-related pathway scores using the ssGSEA method in malignant cells and non-malignant cells. Higher scores of senescence-related pathways were found in malignant cells than that of non-malignant cells (*p* < 0.0001) (Figure 2C).

### Cellular senescence was verified based on bulk RNA-seq data

**FIGURE 3:**
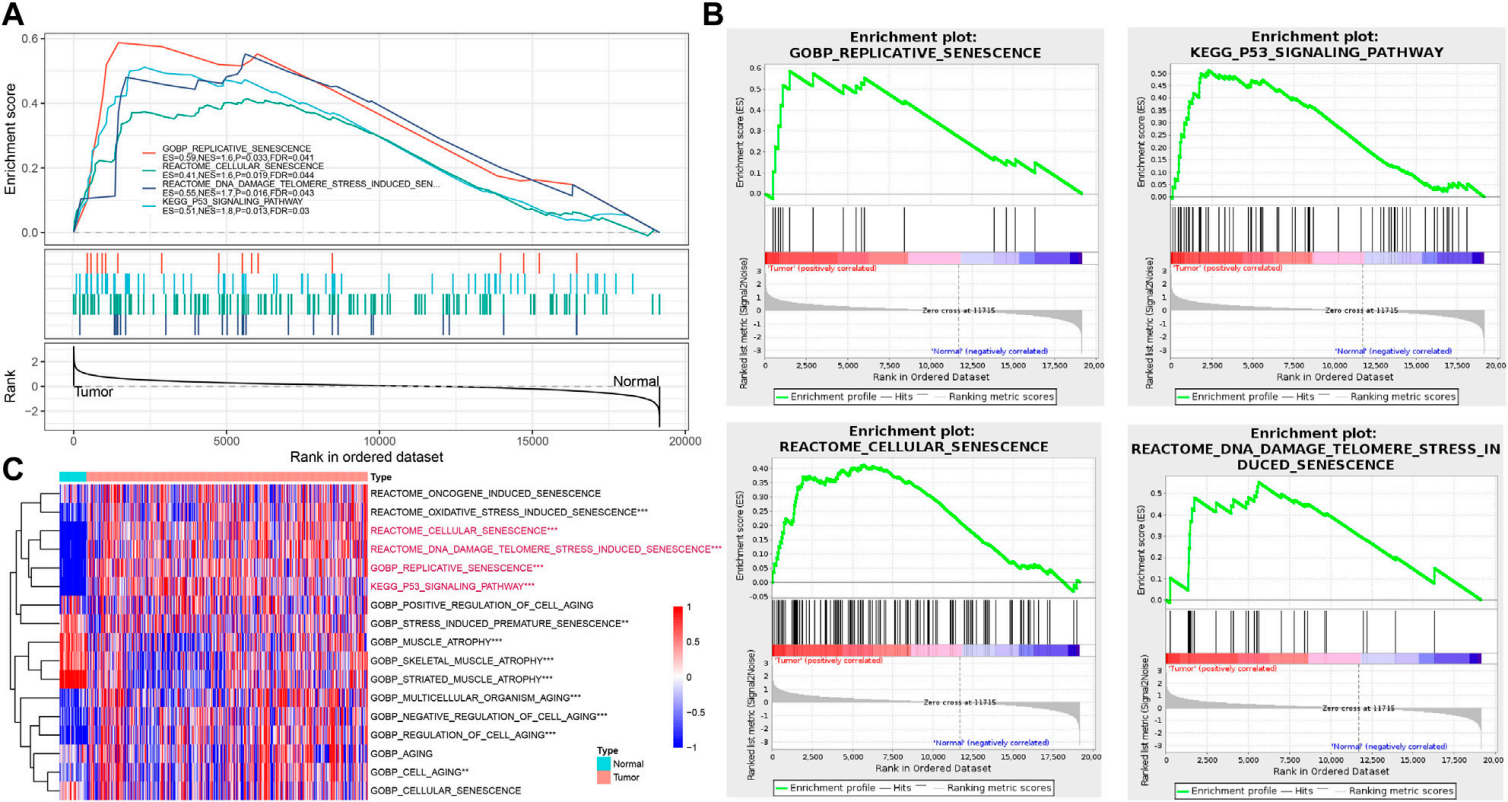
Verification of cellular senescence based on bulk RNA-seq data. **(A)**, The results of GSEA enrichment analysis in TCGA cohort. **(B)**, Four key pathways of GSEA analysis in TCGA cohort. **(C)**, Heatmap of ssGSEA scores of senescence-related pathways between tumor and para-cancer tissues in TCGA cohort. ^*^ *p* < 0.05, ^**^ *p* < 0.01, and ^***^ *p* < 0.001.

To further verify the cellular senescence characteristics, we evaluated the senescence-related pathways in tumor and para-cancer tissues based on bulk RNA-seq data. GOBP_REPLICATIVE_SENESCENCE, REACTOME_CELLULAR_SENESCENCE, REACTOME_DNA_DAMAGE_TELOMERE_STRESS_INDUCED_SENESCENCE, and KEGG_P53_SIGNALING_PATHWAY were significantly enriched in tumor tissues in TCGA cohort (Figures 3A, B). Through calculating senescence-related pathway scores using the ssGSEA method, several pathways including RGOBP_REPLICATIVE_SENESCENCE, REACTOME_CELLULAR_SENESCENCE, REACT OME_DNA_DAMAGE_TELOMERE_STRESS_INDUCED_SENES CENCE, and KEGG_P53_SIGNALING_PATHWAY had higher senescence scores in tumor tissues than that of para-cancer tissues (*p* < 0.001) (Figure 3C).

### Three senescence subtypes were identified

**FIGURE 4:**
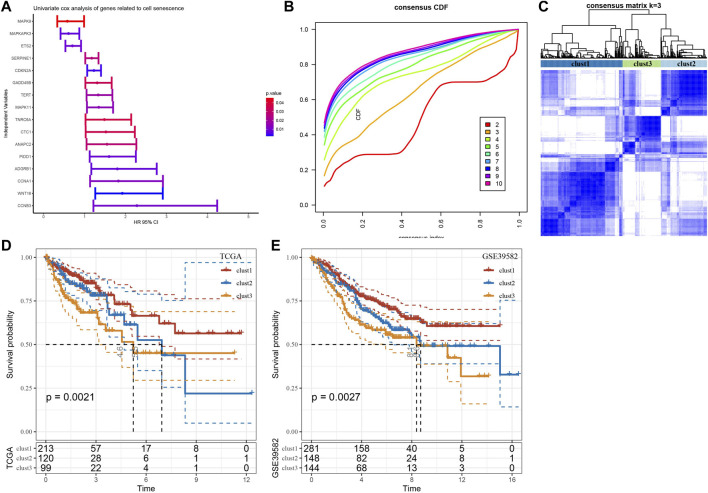
Three senescence subtypes were identified. **(A)**, Univariate cox regression analysis of senescence-related genes. **(B)**, Consensus CDF in TCGA cohort. **(C)**, Consensus matrix heatmap defining three clusters (k = 3). **(D-E)**, Kaplan-Meier curves of three subtypes in TCGA cohort and in GSE39582 cohort.

We performed univariate Cox regression analysis using genes from these four enriched pathways above (Supplementary Table S1). A total of 16 genes associated with prognosis were identified (Figure 4A). To further identify the subtypes, a consensus clustering analysis was conducted to categorize the 432 TCGA-COAD samples based on the expression profiles of the 16 senescence-related genes. From the results of CDF Delta area, cluster = 3 had a relatively stable clustering effect (Figure 4B). Considering that consensus matrix k = 3 is a preferable choice, we divide the whole cohort into three subtypes (Figure 4C). Next, Kaplan-Meier curves revealed significant variations among the three subtypes, and clust3 had the lowest survival probability while clust1 had the best prognosis in TCGA (*p* = 0.0021) (Figure 4D). Similar results were also observed in GSE39582 cohort (*p* = 0.0027) (Figure 4E).

### Clust3 had poorest prognosis and higher T stage

**FIGURE 5:**
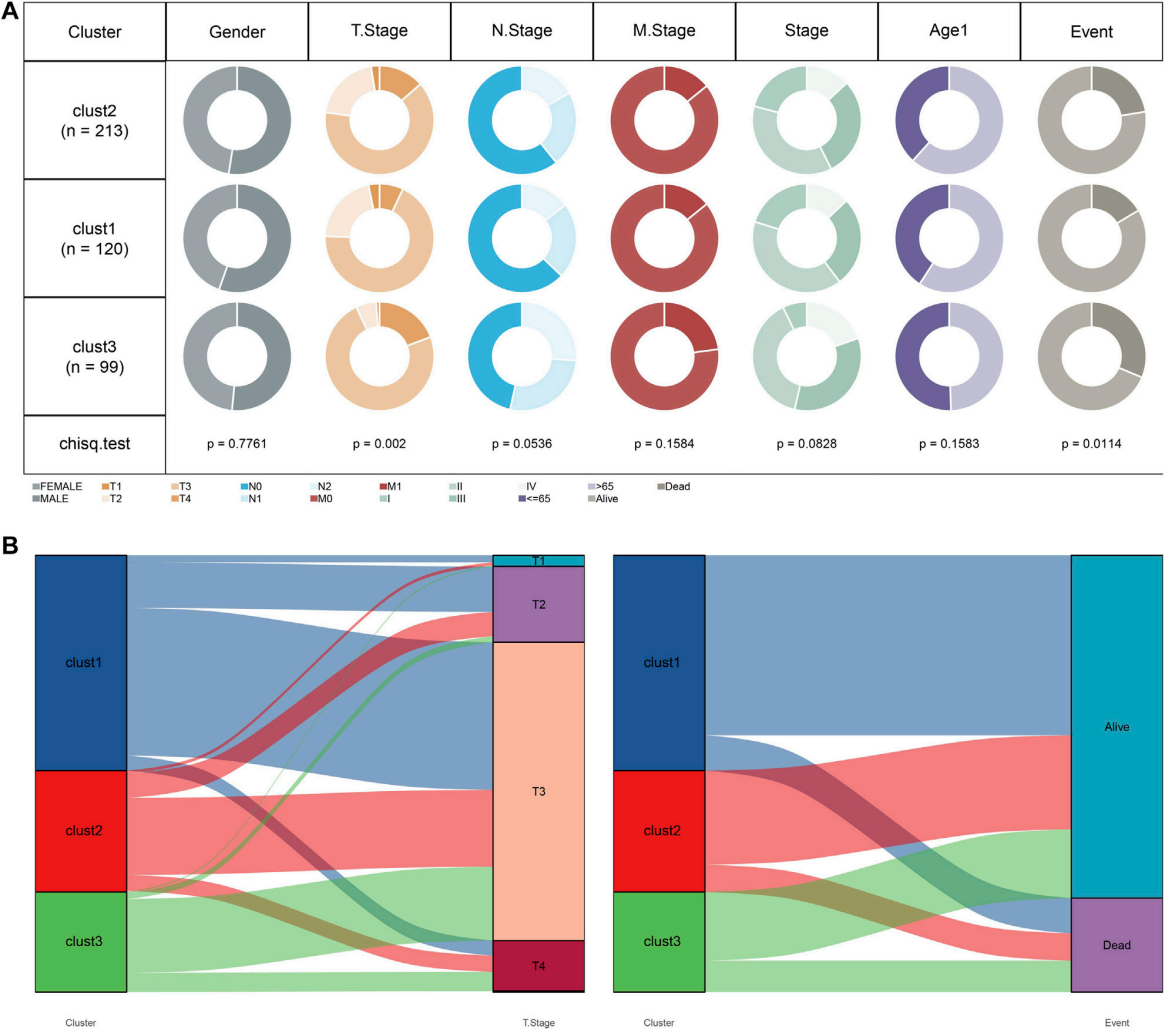
Clust3 had poorest prognosis and higher T stage. **(A)**, Distribution of clinicopathologic characteristics among three subtypes in TCGA cohort. **(B)**, Sankey diagram detailed the distribution of three subtypes in T stage and survival status.

We subsequently compared the distribution of clinicopathologic characteristics (gender, T stage, N stage, M stage, Stage, age, and survival status) among three subtypes in TCGA cohort. The results found significant differences in T stage and survival status among the three subtypes (Figure 5A). Additionally, Sankey diagram detailed the distribution of three subtypes in T stage and survival status (Figure 5B). The patients with clust3 had poorest prognosis and higher T stage (predominantly in T3 and T4 stage).

### Clust3 exhibited higher TMB and mutations

**FIGURE 6:**
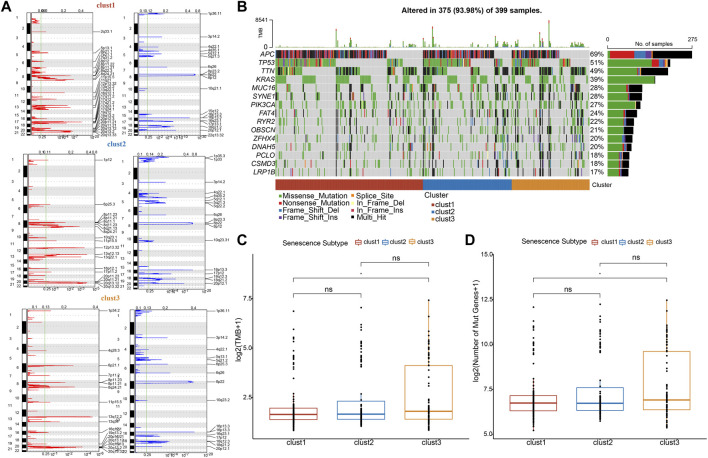
Clust3 exhibited higher TMB and mutations. **(A)**, Peaks of CNVs, amplified (red) genes and deleted (blue) genes among three subtypes. **(B)**, Top 15 mutated SNV genes among three subtypes. **(C)**, TMB alterations among three subtypes. **(D)**, The number of mutated genes among three subtypes. Ns represents *p* > 0.05; ^*^ *p* < 0.05.

Furthermore, mutation characteristics were further evaluated among senescence subtypes. The CNVs were remarkably changed among three subtypes (Figure 6A). Meanwhile, the results from SNVs showed APC (69%), TP53 (51%), TTIN (49%), and KRAS (39%) exhibited higher mutation frequencies among top 15 mutated genes (Figure 6B). Besides, TMB and the number of mutated genes were both increased in clust3 compared with that of clust1 (Figures 6C, D).

### Senescence subtypes were associated with EMT, hypoxia, angiogenesis and tumor-related pathways

**FIGURE 7:**
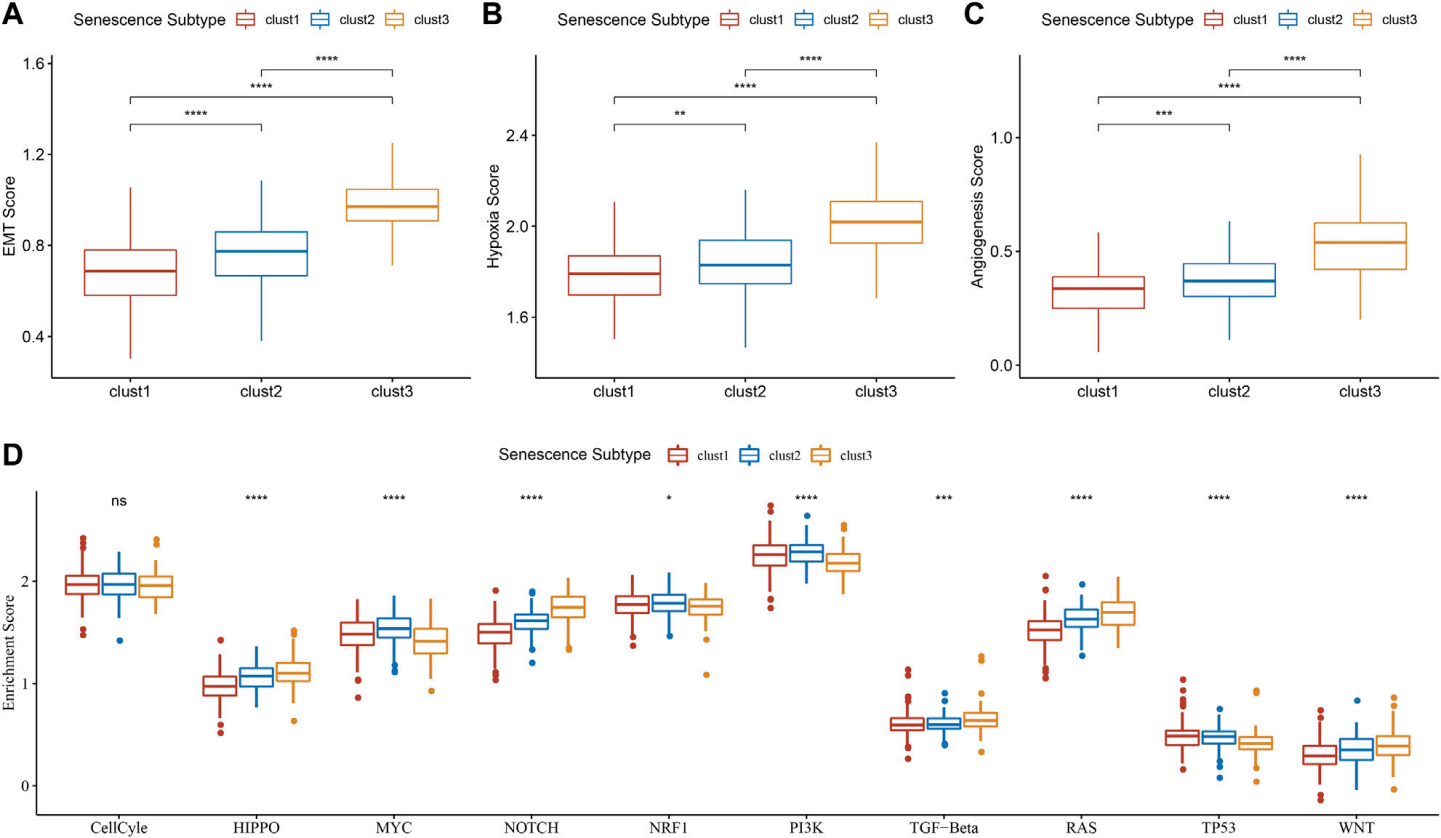
Association of senescence subtypes between EMT, hypoxia, angiogenesis and tumor-related pathways. **(A)**, Box plots of EMT score among three subtypes in TCGA cohort. **(B)**, Box plots of hypoxia score among three subtypes in TCGA cohort. **(C)**, Box plots of angiogenesis score among three subtypes in TCGA cohort. **(D)**, Box plots of 10 tumor-related pathways among three subtypes in TCGA cohort. Ns represents *p* > 0.05; ^*^ *p* < 0.05, ^**^ *p* < 0.01, ^***^ *p* < 0.001, and ^****^ *p* < 0.0001.

In tumors, cellular senescence promotes the extracellular matrix cleavage resulting in growth factors release that can promote epithelial-to-mesenchymal transition (EMT), which leads to tumor metastasis. Hence, we clarified the relationship between senescence subtypes and EMT score. EMT score was distinctly different among senescence subtypes and clust3 has the highest EMT score compared with that of clust1 and clust2 (Figure 7A). At the same time, hypoxia and angiogenesis scores were higher in clust3 than that of clust1 and clust2 (Figures 7B, C). Figure 7D found that 9 tumor-related pathways were significantly altered in the three subtypes, including cell cycle, HIPPO, MYC, NOTCH, NRF1, PI3K, TGF-beta, RAS, TP53 and WNT.

### Relationship between senescence subtypes and immune characteristics

**FIGURE 8:**
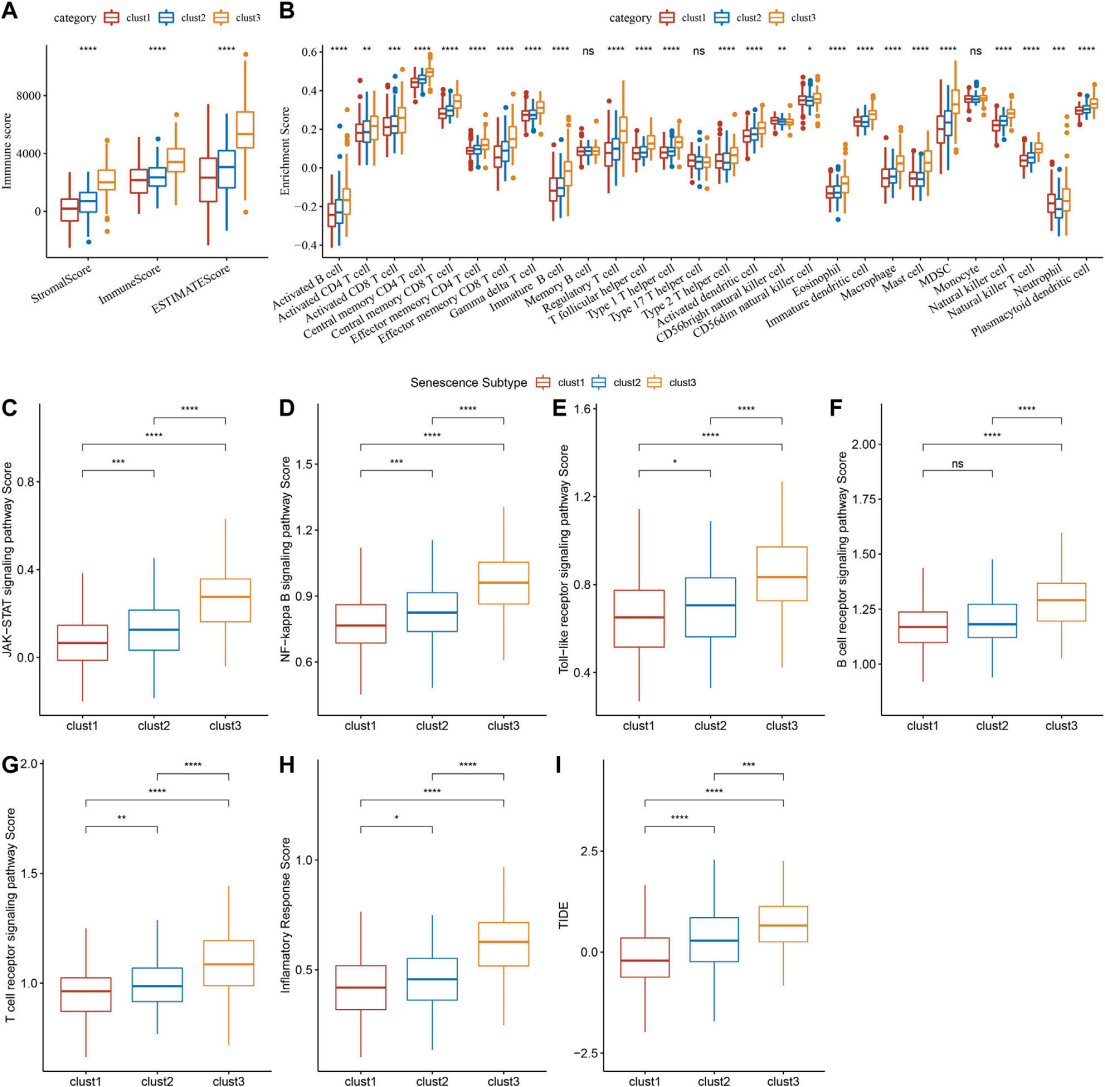
Association of senescence subtypes with immune characteristics. **(A)**, Box plots of immune infiltration scores among three subtypes. **(B)**, Box plots of immune infiltration cells among three subtypes. **(C–H)**, Box plots of JAK-STAT signaling pathway, NF-kappa B signaling pathway score, toll-like receptor signaling pathway, B cell receptor signaling pathway, T cell receptor signaling pathway and inflammatory response scores among three subtypes. **(I)**, Alteration of TIDE score among three subtypes. Ns represents *p* > 0.05; ^*^ *p* < 0.05, ^**^ *p* < 0.01, ^***^ *p* < 0.001, and ^****^ *p* < 0.0001.

Immune infiltration scores including StromalScore, ImmuneScore, and ESTIMATEScore were remarkably different among three subtypes (Figure 8A), and we found that clust3 had a higher degree of immune infiltration. Significant changes in immune cells infiltration were found among the three subtypes (Figure 8B). Clust3 also had higher scores of several inflammation-related pathways such as JAK-STAT signaling pathway, NF-Kappa B signaling pathway, Toll-like receptor signaling pathway, B cell receptor signaling pathway, T cell receptor signaling pathway, and inflammatory response (Figures 8C–H). Furthermore, TIDE score was higher in clust3 (Figure 8I), indicating more prone to immune escape of clust3.

### Construction and validation of senescence-based risk model

**FIGURE 9:**
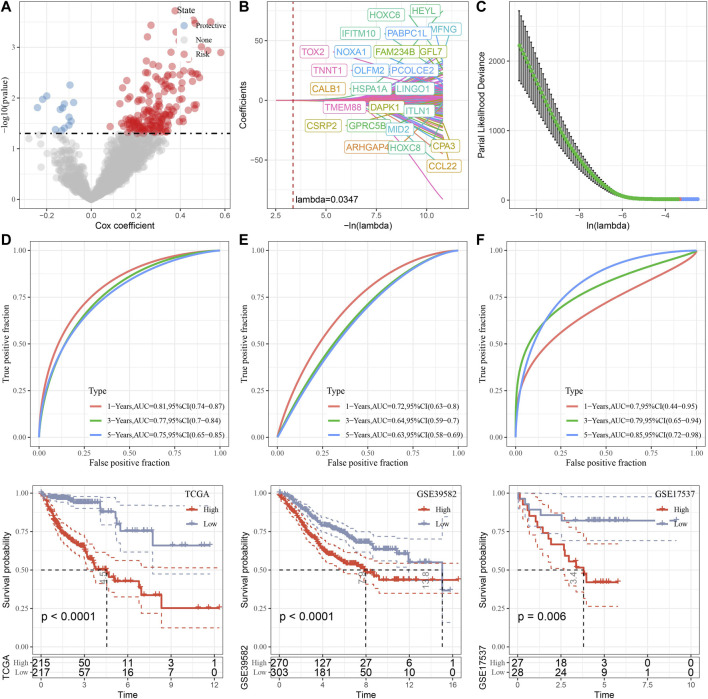
Construction and validation of senescence-based risk model. **(A)**, A total of 2,085 promising candidates were identified among the DEGs. **(B)**, Independent variable coefficients changed with lambda increase. When lambda = 0.00347, 25 genes were identified. **(C)**, 10-fold cross validation to determine the confidence interval under each lambda. **(D–F)**, Survival analysis with ROC curves and Kaplan-Meier curves in TCGA cohort, GSE39582 cohort and GSE17537 cohort.

Through differential analysis among the three subtypes, 2,085 DEGs were identified, which were used for univariate Cox regression analysis. 194 genes that have greater impact on prognosis were selected, including 180 risk genes and 14 protective genes (Figure 9A). To reduce the number of genes, LASSO Cox regression was performed. With the gradual increase of lambda, the number of independent variable coefficients tending to zero increased gradually (Figure 9B). 10-fold cross-validation was utilized and the confidence interval under each lambda was shown in (Figure 9C). When lambda = 0.0347, 25 genes were selected for further analysis. Based on stepwise multivariate regression analysis with AIC, 11 genes were finally identified (MFNG, GPRC5B, TNNT1, CCL22, NOXA1, PABPC1L, PCOLCE2, MID2, CPA3, HSPA1A, and CALB1). Subsequently, survival analysis in TCGA cohort revealed that patients with high risk had lower prognosis than that of patients with low risk (*p* < 0.0001) with 1 year AUC of 0.81, 3-year AUC of 0.77, and 5-year AUC of 0.75 (Figure 9D). To validate its robustness, survival analysis was performed in GSE39582 and GSE17537 cohort. High risk patients had lower prognosis than that of low risk patients in GSE39582 (*p* < 0.0001) and in GSE17537 (*p* = 0.006) with good performance in prognosis prediction (Figures 9E, F).

### Associations of risk score with clinicopathologic characteristics and biological characteristics

**FIGURE 10:**
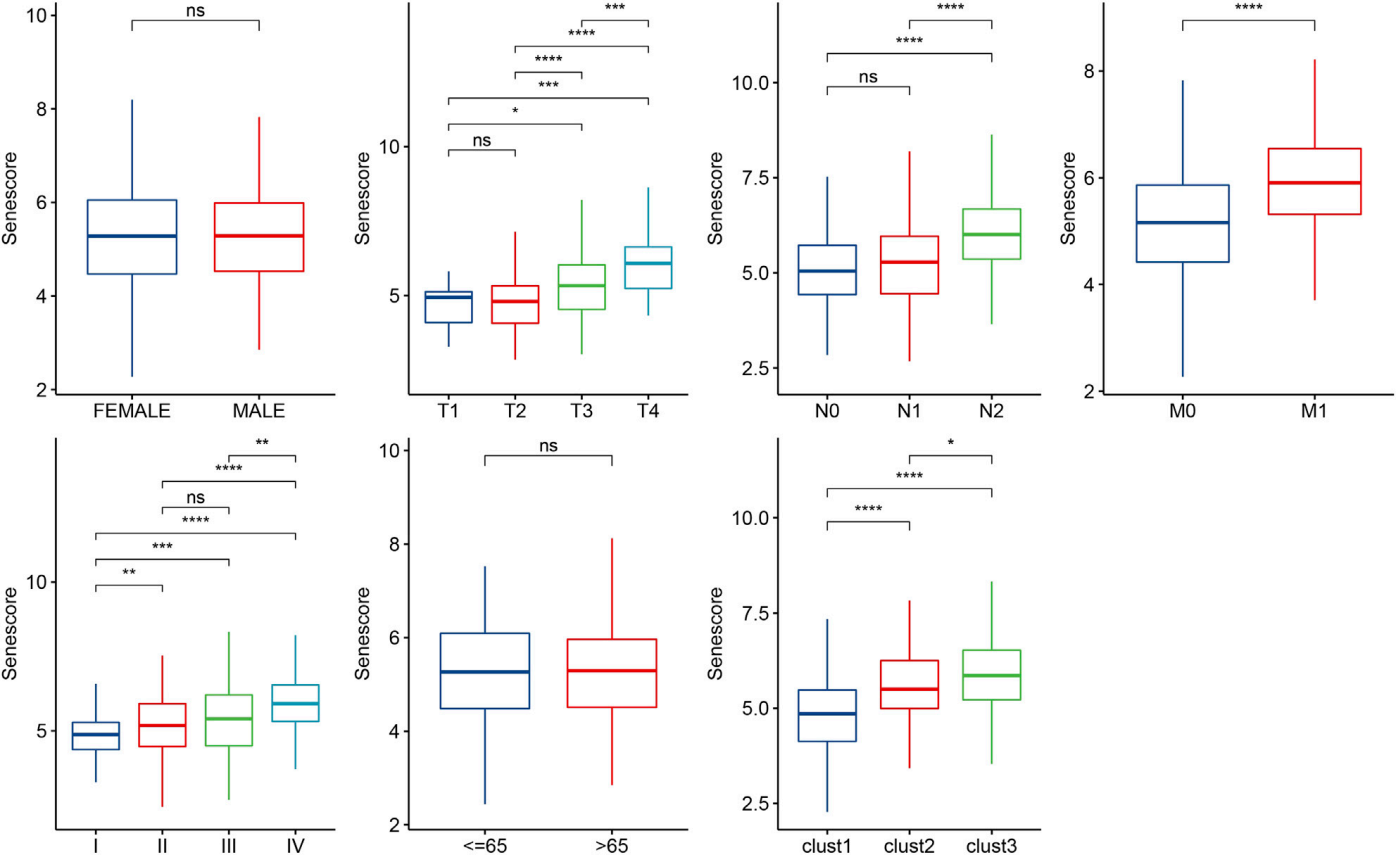
Distribution of risk score in different clinicopathologic characteristics. Ns represents *p* > 0.05; ^*^ *p* < 0.05, ^**^ *p* < 0.01, ^***^ *p* < 0.001, and ^****^ *p* < 0.0001.

**FIGURE 11:**
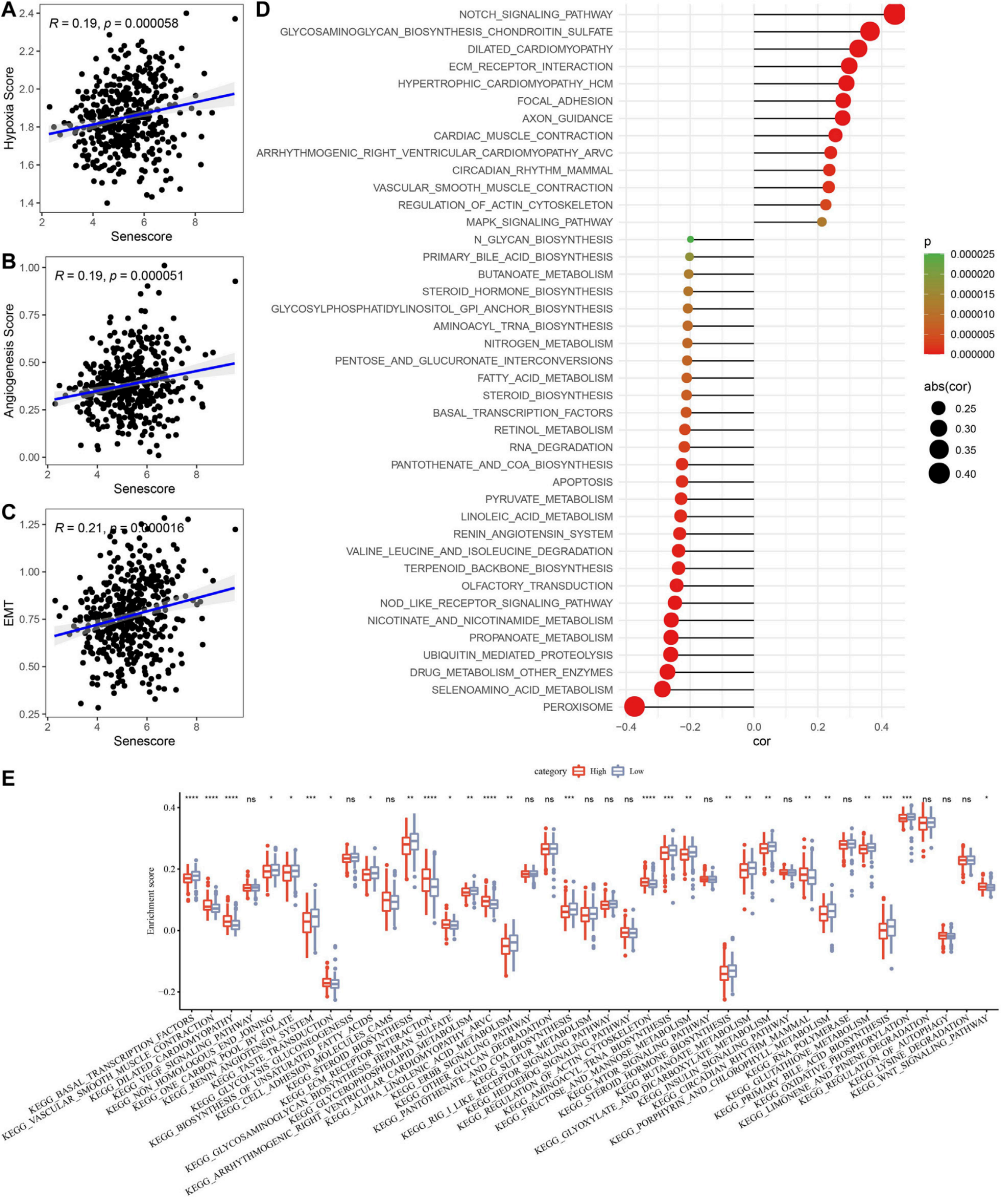
Associations of risk score with biological characteristics. **(A–C)**, Correlation analysis of risk score with hypoxia, angiogenesis, and EMT scores. **(D)**, Scatter plots of correlation between risk score and underlying regulatory KEGG pathways. **(E)**, Box plots of underlying regulatory KEGG pathway scores between high- and low-risk group. Ns represents *p* > 0.05; ^*^ *p* < 0.05, ^**^ *p* < 0.01, ^***^ *p* < 0.001, and ^****^ *p* < 0.0001.

To further clarify the relationship between risk score and clinicopathologic characteristics, we compared the differences of risk score in clinicopathologic characteristics in TCGA cohort and found that patients with higher clinical stage (T stage, N stage, M stage and Stage) had higher risk scores (Figure 10). Besides, patients with clust3 had higher risk score (Figure 10). To evaluate the relationship between risk score and biological characteristics, we performed the correlation analysis of risk score with hypoxia, angiogenesis, and EMT scores. Figures 11A–C showed that hypoxia, angiogenesis, and EMT scores were both positively correlated with risk score. Next, we performed correlation analysis between senescence-based risk score and underlying regulatory KEGG pathways to find risk score-related pathways (Figure 11D) and further statistics revealed that only some pathways were significant different between high- and low-risk group (Figure 11E). Moreover, we compared the scores of senescence-related pathways between high- and low risk groups. The results showed that high risk patients exhibited higher scores in GOBP_AGING, GOBP_MUSCLE_ATROPHY, and GOBP_NEGATIVE_REGULA TION_OF_CELL_AGING; while low risk patients had higher scores in GOBP_STRESS_INDUCED_PREMATURE_SENESC ENCE, KEGG_P53_SIGNALING_PATHWAY, and REACTO ME_DNA_DAMAGE_TELOMERE_STRESS_INDUCED_SENE SCENCE (Supplementary Figure S3).

### Prediction of responsiveness to chemotherapy

**FIGURE 12:**
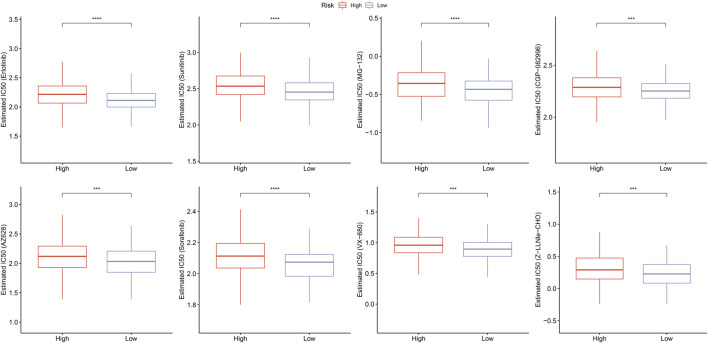
Prediction of responsiveness to chemotherapy. Estimated IC50 values for traditional chemotherapy drugs including erlotinib, sunitinib, MG-132, CGP-082996, AZ628, sorafenib, VX-680, and Z-LLNle-CHO. ^***^ *p* < 0.001, and ^****^ *p* < 0.0001.

Furthermore, we assessed the responsiveness to traditional chemotherapy drugs between high- and low-risk groups. As displayed in Figure 12, low risk patients exhibited significant lower IC50 values of Erlotinib (*p* < 0.001), Sunitinib (*p* < 0.001), MG-132 (*p* < 0.001), CGP-082996 (*p* < 0.01), AZ628 (*p* < 0.01), Sorafenib (*p* < 0.001), VX-680 (*p* < 0.01), and Z-LLNle-CHO (*p* < 0.01) than that of high risk patients, which indicated that low risk patients were more sensitive to Erlotinib, Sunitinib, MG-132, CGP-082996, AZ628, Sorafenib, VX-680, and Z-LLNle-CHO.

### Relationship between risk gene expression and methylation level

**FIGURE 13:**
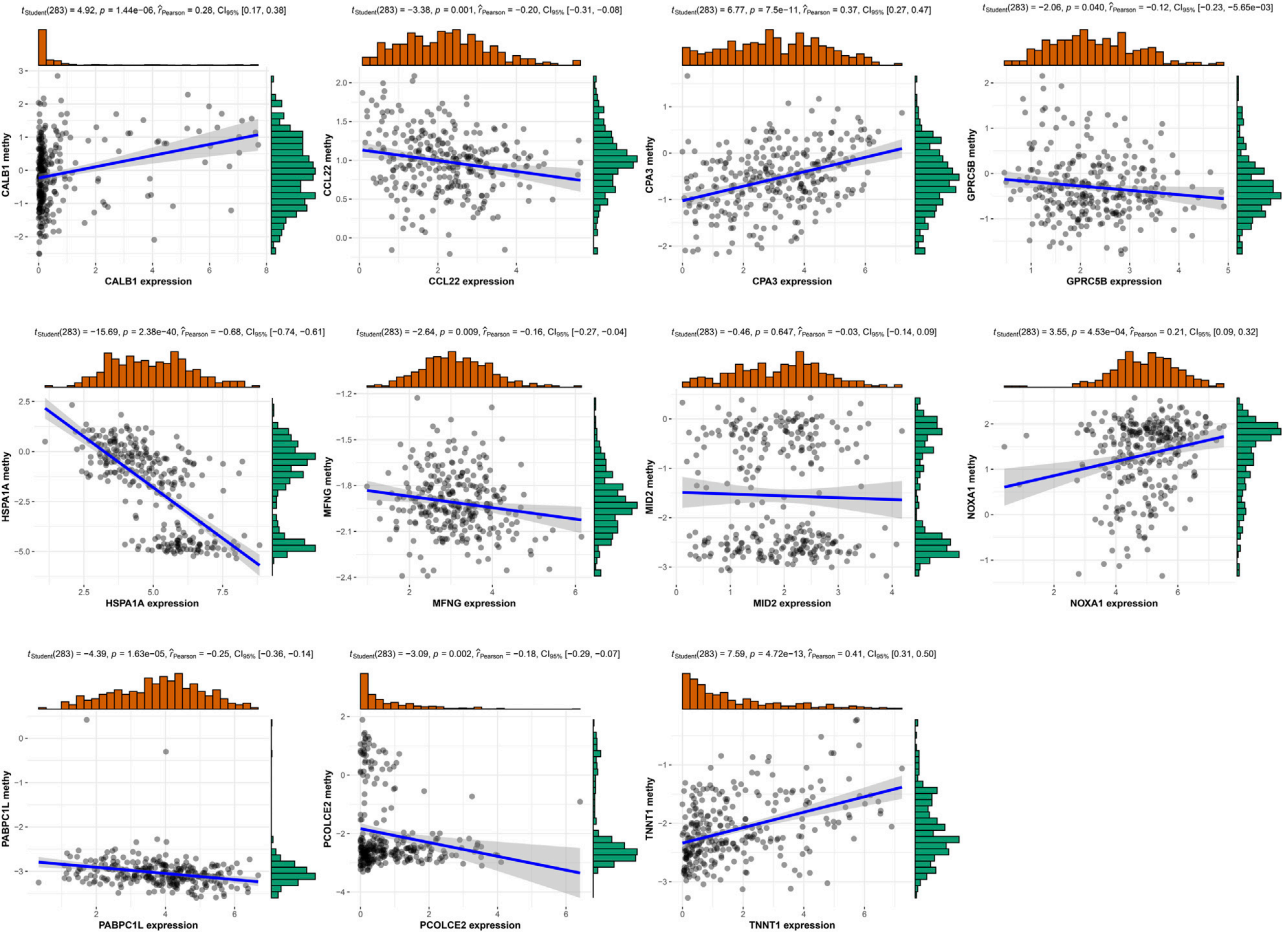
Correlation analysis was performed between risk gene expression and methylation level.

Moreover, we analyzed the methylation level for 11 risk genes in TCGA (Supplementary Figure S4) and performed correlation analysis between risk gene expression and methylation level. Figure 13 displayed that the expression of CALB1, CPA3, NOXA1, and TNNT1 had significant positive correlation with their methylation level; whereas the expression of CCL22, GPRC5B, HSPA1A, MFNG, PABPC1L, and PCOLCE2 had significant negative correlation with their methylation level.

### Construction of nomogram

**FIGURE 14:**
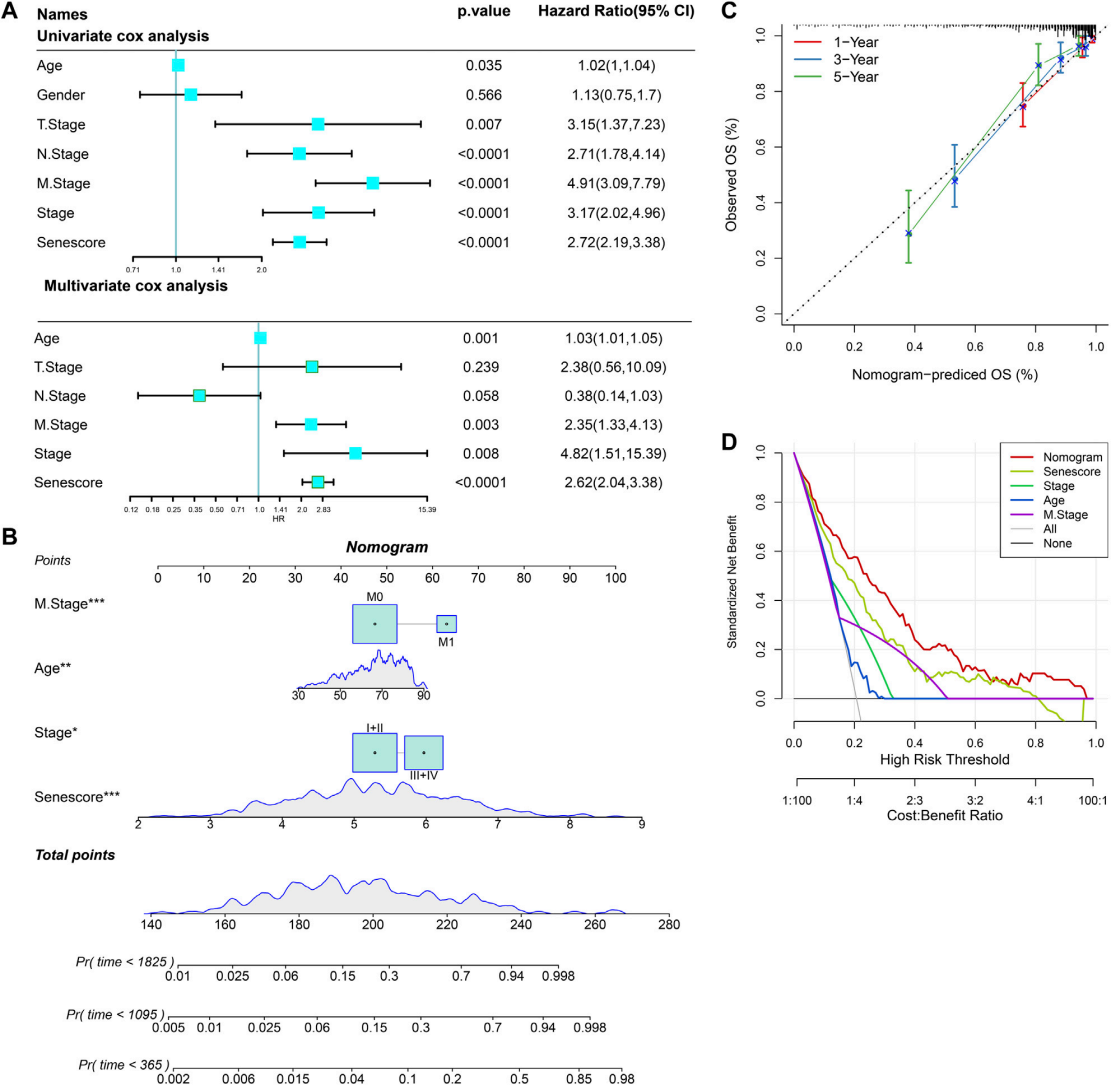
Nomogram construction for predicting prognosis of COAD patients. **(A)**, Univariate and multivariate Cox regression analysis of prognostic values of risk score and clinicopathological characteristics. **(B)**, Nomogram for predicting the 1-, 3-, and 5-year OS of COAD patients. **(C)**, Calibration curves for validating the established nomogram. **(D)**, Decision curve analysis o of nomogram. ^*^ *p* < 0.05, ^**^ *p* < 0.01, and ^***^ *p* < 0.001.

On basis of univariate and multivariate Cox regression analysis, the results showed that risk score was an independent prognostic factor (Figure 14A). To better quantify the risk assessment and survival probability of COAD patients, we constructed a nomogram to estimate 1-, 3-, and 5-year OS using the risk score and clinicopathological characteristics. Figure 14B revealed that risk score had the most impact on OS of COAD patients. The calibration curves of this nomogram showed high consistency between the observed and predicted values (Figure 14C). To evaluate the reliability of this model, DCA analysis was conducted and confirmed that both nomogram and risk score had the most powerful in predicting prognosis (Figure 14D).

## Discussion

Senescent cells are closely related to aging and pathological status. Cellular senescent is crucial in tumorigenesis through the SASPs and the heterogeneity of senescence-associated genes promotes the progression of tumor and its escape from anti-tumor therapy (Junaid et al., 2022). Thus, it is believed that cellular senescence is involved in cancer heterogeneity. A previous study has identified several senescence-associated gene signatures using transcriptome data from TCGA database, which can predict clinical outcomes and responses to immunotherapy in patients with head and neck squamous cell carcinoma (Wang et al., 2022). Another study has developed the senescence-related subtypes, established a prognostic risk model, and further revealed their potential roles in TME in breast cancer only using transcriptome data from GEO cohort (Zhou et al., 2022). In the present study, we downloaded the scRNA-seq data of 13 COAD samples from GEO database and demonstrated that senescence-related pathways were highly expressed in malignant cells than that of non-malignant cells, indicating that cellular senescence was largely associated with heterogeneity of TME in COAD at single cell level. Furthermore, we identified three senescence subtypes and found clust3 had poorest prognosis, manifested with higher T stage, elevated TMB, increased pathway scores (EMT, hypoxia and angiogenesis), activated inflammatory response, and immune cell infiltration as well as immune escape tendency.

Immune cell infiltration serves as an indicator of the immune microenvironment in tumor. Notably, we found that clust3 patients with poor prognosis had extremely higher expression levels of myeloid-derived suppressor cells (MDSCs) and macrophages than that of other subtypes. It has been reported that MDSCs are immature myeloid cells with heterogeneity, and its accumulation suppress anti-tumor immunity particular suppressing T cells in cancer patients (Ma et al., 2019). Additionally, MDSCs also directly promote tumor growth and metastasis. MDSCs exert these effects mainly through inhibiting T cell proliferation and T cell migration, triggering apoptosis of T cells and NK cells, suppressing immune effector cell functions, and repressing anti-tumor T cell-mediated reactivity by interaction with PD-1 receptor (Umansky et al., 2016). Moreover, macrophage infiltration in solid tumors accounts for poor outcomes and correlates with chemotherapy resistance in most cancers, which contributes to development and progression of cancer *via* provoking angiogenesis, metastasis, and immunosuppression (Cassetta and Pollard, 2018). In this study, highly expressed MDSCs might exert inhibitory effects on T cells and NK cells, inducing a decline of immune function in TME of COAD patients. Besides, macrophage infiltration might induce the suppression of immunity. We also found that patients with poor prognosis had a high TIDE score that represented a low response rate to ICI therapy. Collectively, MDSC infiltration, macrophage infiltration and low response rate to ICI therapy contribute to poor clinical outcome of COAD patients in clust3. Synergistically, EMT, hypoxia, angiogenesis, and activated inflammatory response were responsible for poor prognosis of COAD patients.

Highly variant tumors are considered to have an increased burden of new antigens that may lead to immunogenicity. It has been recognized that TMB is a potential immune-response marker predicting ICI therapy (Choucair et al., 2020). As a tumor suppressor gene, APC is highly mutated in CRC (about 70%), and its mutation is important in colorectal tumorigenesis (Zhang and Shay, 2017). Recently, a study has analyzed the clinical characterizes and gene mutations in APC-mutant type and APC-wild-type Chinese CRC patients and confirms that APC mutation can be used as a promising biomarker to predict the immunotherapy responsiveness (Feng et al., 2022b). Another tumor suppressor gene TP53, is thought to be a major driver for CRC with approximately 50% mutation frequency (Timar and Kashofer, 2020). It has been reported that TP53 mutation is closely associated with rectum tumor, advanced stage and dismal prognosis of CRC patients (Li, 2019). Additionally, RAS is the most frequent mutated gene in human cancers. Interestingly, KRAS and APC are very common co-mutated (about 80%) and co-mutation of KRAS with TP53 is about 40% in CRC (Timar and Kashofer, 2020). In this study, APC (69%), TP53 (51%), TTIN (49%), and KRAS (39%) exhibited higher mutation frequencies among top 15 mutated genes. The TMB and the number of mutated genes were both increased in clust3 than that of clust1. These results indicated that the higher TMB and large mutations contributed to poor prognosis of COAD patients. Notably, we found that TTIN (49%) was highly mutated except for the known APC, TP53, and KRAS, implying that TTIN mutation is a potential genetic alteration of COAD heterogeneity.

Furthermore, 11-senescence-related gene-based prognostic risk model was established and patients with high risk had lower prognosis. MFNG is a kind of glycosyltransferases that activates Notch signaling and plays an important role in breast cancer, whereas inhibition of MFNG may attenuate the triple-negative breast cancer (Mugisha et al., 2022). A whole transcriptomics analysis has revealed that GPRC5B is elevated in immuno-activated breast cancer cells, while apigenin induces a 94% reduction in GPRC5B expression (Bauer et al., 2019). A recent research of RNA-seq has showed TNNT1 is a representative prognostic mRNAs that is associated with the prognosis of CRC patients (Deng et al., 2022). It has confirmed that high expression of CCL22 is related to a better prognosis in patients with colon cancer, and CCL22 as a prognostic DEG is used to construct a cellular senescence-related risk model in colon cancer (Dai et al., 2022b). NADPH oxidase 1 (NOX1), derived reactive oxygen species and modulated by NOXA1, is crucial in the progression of cancer (Attri et al., 2020). PABPC1L is a key gene in tumor progression and postoperative prognosis, while inhibition of PABPC1L suppresses CRC cell growth and metastasis (Wu et al., 2019). Meanwhile, PABPC1 and FOXC2 bind to cis-regulatory elements and inhibit cellular senescence through downregulating p16INK4a in endothelial cells (Wu et al., 2022). Similarly, PCOLCE2 is a novel senescence-related gene that is used to establish a prognostic model in CRC (Yao et al., 2021). MID2, as a promoter of STAT3, is interacted with protein MORC4, which regulates DNA damage response and gene transcription in breast cancer (Wang et al., 2021). CPA3 belongs to carboxypeptidase family of zinc metalloproteases released by mast cells and has been demonstrated to be involved in endogenous proteins degradation as well as colon cancer prognosis (Fang et al., 2021). The 70 kDa heat shock protein, called HSPA1A, is considered as a potential biomarker for the initiation and development breast cancer (de Freitas et al., 2022). The level of HSPA1A is upregulated after heat stress response, but is downregulated by senescence (Llewellyn et al., 2021). It has been reported that CALB1 can promote the interaction between p53 and MDM2, and alleviates ovarian cancer cell senescence (Cao et al., 2019). Collectively, these results suggest that the prognostic genes in senescence-based signatures may be crucial in cellular senescence and prognosis of COAD.

There are some limitations in this study. Data from TCGA and GEO are collected and used for bioinformatics analysis in this study, and these retrospective data may have selection bias. Thus, prospective studies with large samples are needed to validate these results. Although the robustness of our prognostic risk model has been validated by external GEO datasets, its reliability should be iteratively improved with long-term clinical application. Besides, the regulatory mechanism of MDSCs and macrophages on T cells and NK cells should be further investigated in COAD patients with poorer prognosis and better prognosis according to these senescence-based subtypes.

## Conclusion

In conclusion, we developed senescence-based subtypes that could distinguish prognosis, T stage, mutation and immune characteristics, which might guide further mechanism investigation of heterogeneity of COAD. Additionally, we constructed and validated a senescence-based signature and provided a reliable tool for prognosis prediction in COAD patients.

## Data Availability

The original contributions presented in the study are included in the article/Supplementary Material, further inquiries can be directed to the corresponding authors.

## Abbreviations

CRC, colorectal cancer; COAD, colon adenocarcinoma; scRNA-seq, single-cell RNA sequencing; TME, tumor microenvironment; TCGA, the cancer genome atlas; GEO, gene-expression omnibus; PCA, principal component analysis; ICI, immune checkpoint inhibition; TIDE, tumor immune dysfunction exclusion; OS, overall survival; KEGG, kyoto encyclopedia of genes and genomes; CDF, cumulative distribution function; ssGSEA, single-sample gene set enrichment analysis; ROC, receiver operating characteristic analysis; AUC, area under ROC curve; LASSO, least absolute shrinkage and selection operator; AIC, akaike information criterion; EMT, epithelial-to-mesenchymal transition; SNVs, single nucleotide variations; CNVs, copy number variations; TMB, tumor mutation burden; DEGs, differential expressed genes; TSNE, t-distributed stochastic neighbor embedding; PAM, partitioning around medoids; MDSC, myeloid-derived suppressor cells; NOX, NADPH oxidase; DCA, decision curve analysis.
