# Supplementary figures and images for: Comprehensive genomics analysis of aging related gene signature to predict the prognosis and drug resistance of colon adenocarcinoma
Source: Front Pharmacol. 2023 Feb 28;14:1121634. doi: 10.3389/fphar.2023.1121634 (PMC10011090; doi:10.3389/fphar.2023.1121634)

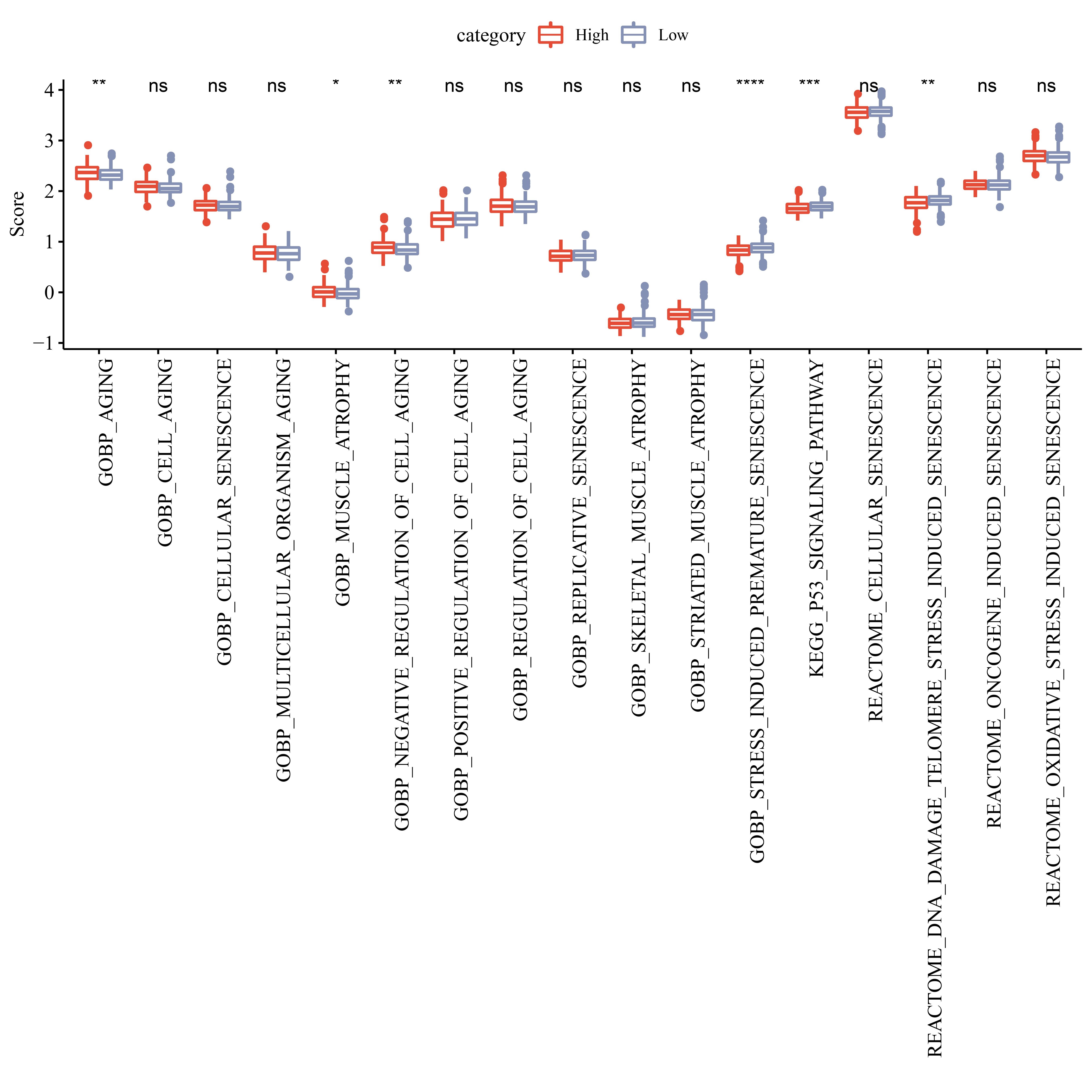

Supplement: Supplementary file 1 [file Image3.JPEG]

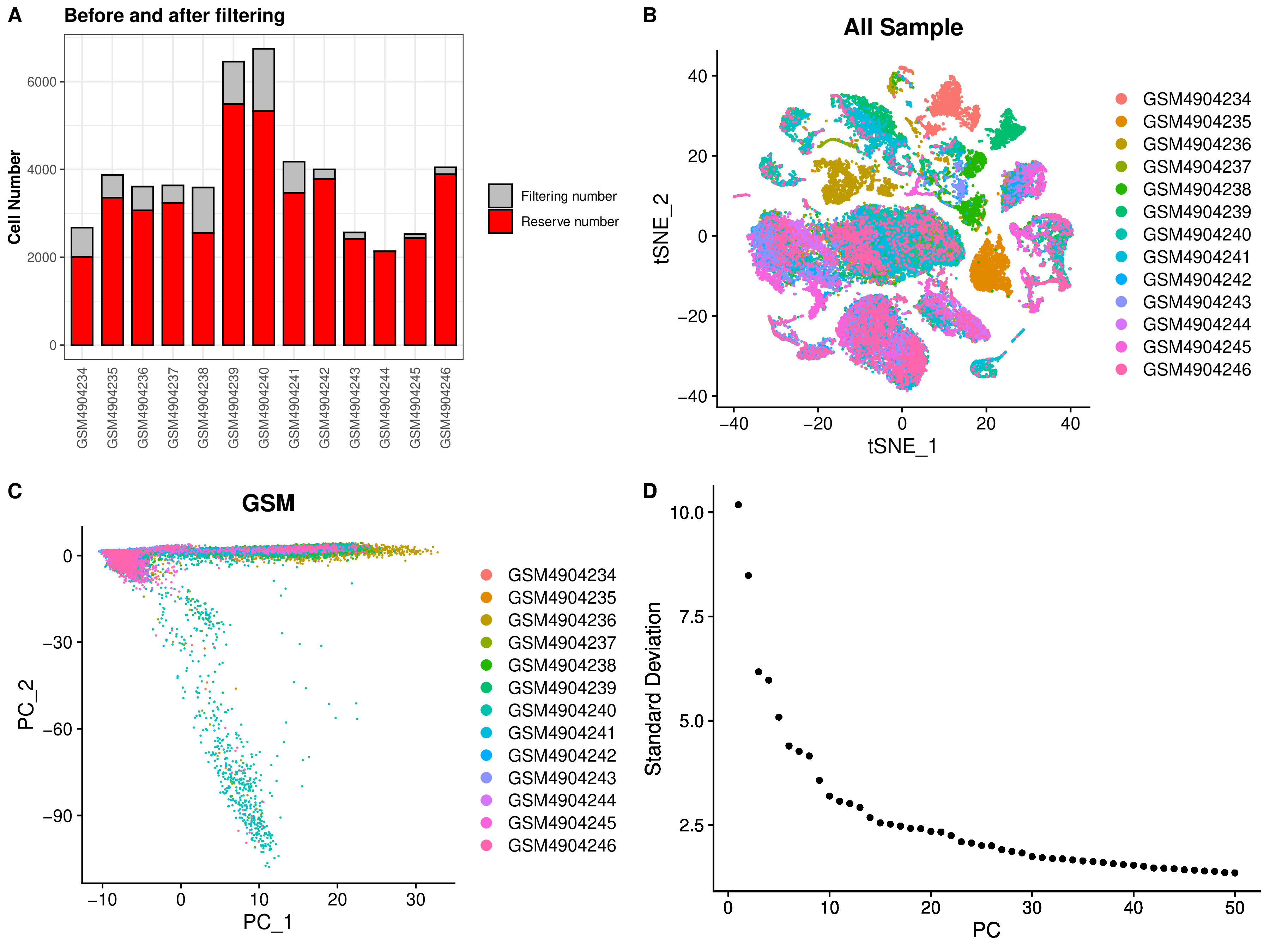

Supplement: Supplementary file 3 [file Image1.JPEG]

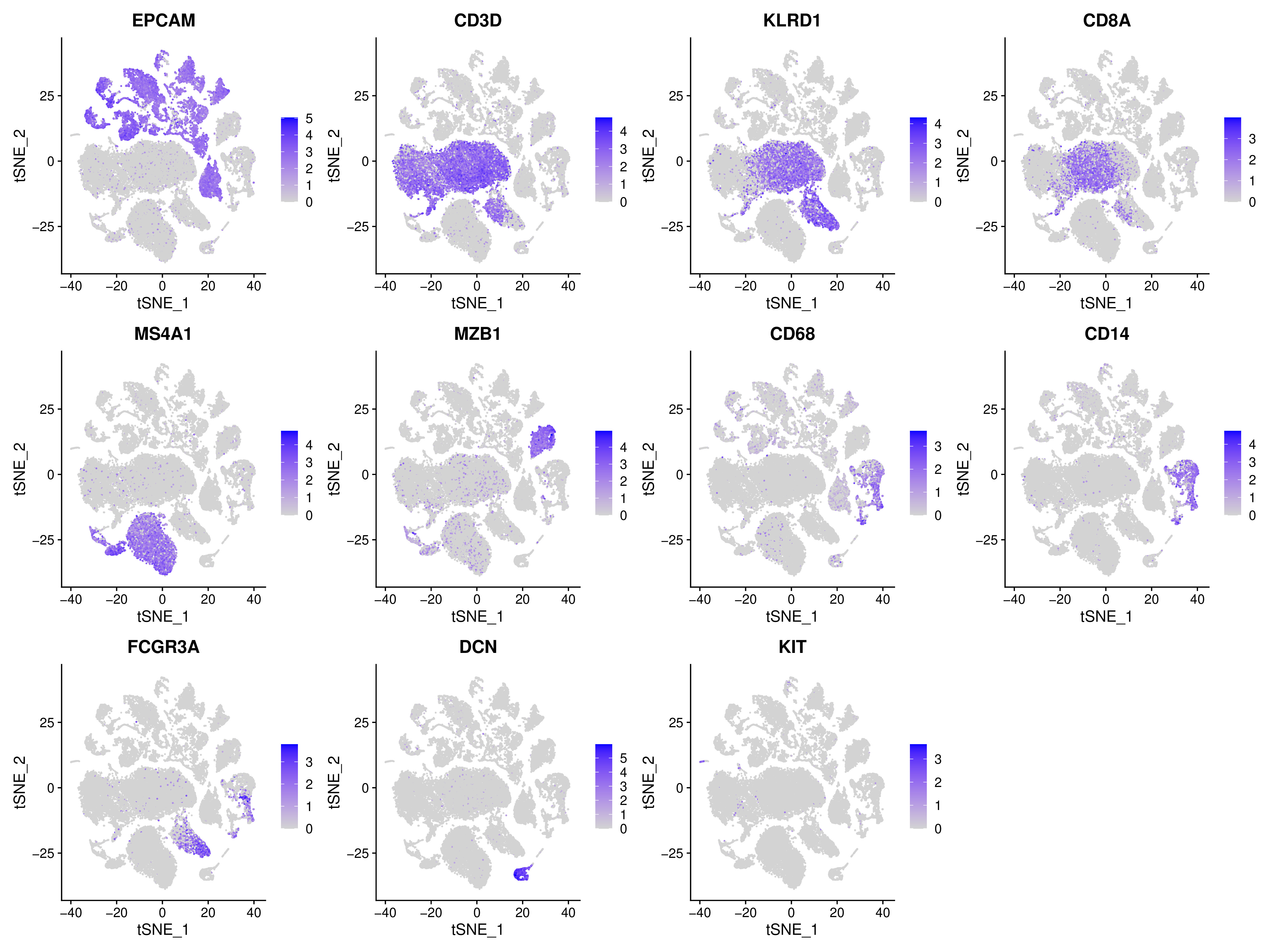

Supplement: Supplementary file 5 [file Image2.JPEG]
