# Supplementary material for: Comprehensive genomics analysis of aging related gene signature to predict the prognosis and drug resistance of colon adenocarcinoma
Source: Front Pharmacol. 2023 Feb 28;14:1121634. doi: 10.3389/fphar.2023.1121634 (PMC10011090; doi:10.3389/fphar.2023.1121634)
Supplement: Supplementary file 2 [file Table1.docx]

**Supplementary Table 1** A total of 262 genes obtained from four senescence-related pathways

|  | Genes from four senescence-related pathways |
| --- | --- |
| 1 | ATM |
| 2 | ATR |
| 3 | CDKN1A |
| 4 | CDKN2A |
| 5 | CHEK1 |
| 6 | CHEK2 |
| 7 | CTC1 |
| 8 | ERCC1 |
| 9 | MIR21 |
| 10 | MME |
| 11 | PLA2R1 |
| 12 | ROMO1 |
| 13 | SERPINE1 |
| 14 | TERT |
| 15 | TP53 |
| 16 | WNT16 |
| 17 | WRN |
| 18 | ADGRB1 |
| 19 | APAF1 |
| 20 | BAX |
| 21 | BBC3 |
| 22 | BID |
| 23 | CASP3 |
| 24 | CASP8 |
| 25 | CASP9 |
| 26 | CCNB1 |
| 27 | CCNB2 |
| 28 | CCNB3 |
| 29 | CCND1 |
| 30 | CCND2 |
| 31 | CCND3 |
| 32 | CCNE1 |
| 33 | CCNE2 |
| 34 | CCNG1 |
| 35 | CCNG2 |
| 36 | CD82 |
| 37 | CDK1 |
| 38 | CDK2 |
| 39 | CDK4 |
| 40 | CDK6 |
| 41 | COP1 |
| 42 | CYCS |
| 43 | DDB2 |
| 44 | EI24 |
| 45 | FAS |
| 46 | GADD45A |
| 47 | GADD45B |
| 48 | GADD45G |
| 49 | GTSE1 |
| 50 | IGF1 |
| 51 | IGFBP3 |
| 52 | MDM2 |
| 53 | MDM4 |
| 54 | PERP |
| 55 | PIDD1 |
| 56 | PMAIP1 |
| 57 | PPM1D |
| 58 | PTEN |
| 59 | RCHY1 |
| 60 | RPRM |
| 61 | RRM2 |
| 62 | RRM2B |
| 63 | SERPINB5 |
| 64 | SESN1 |
| 65 | SESN2 |
| 66 | SESN3 |
| 67 | SFN |
| 68 | SHISA5 |
| 69 | SIAH1 |
| 70 | STEAP3 |
| 71 | THBS1 |
| 72 | TNFRSF10B |
| 73 | TP53AIP1 |
| 74 | TP53I3 |
| 75 | TP73 |
| 76 | TSC2 |
| 77 | ZMAT3 |
| 78 | ACD |
| 79 | AGO1 |
| 80 | AGO3 |
| 81 | AGO4 |
| 82 | ANAPC1 |
| 83 | ANAPC10 |
| 84 | ANAPC11 |
| 85 | ANAPC15 |
| 86 | ANAPC16 |
| 87 | ANAPC2 |
| 88 | ANAPC4 |
| 89 | ANAPC5 |
| 90 | ANAPC7 |
| 91 | ASF1A |
| 92 | BMI1 |
| 93 | CABIN1 |
| 94 | CBX2 |
| 95 | CBX4 |
| 96 | CBX6 |
| 97 | CBX8 |
| 98 | CCNA1 |
| 99 | CCNA2 |
| 100 | CDC16 |
| 101 | CDC23 |
| 102 | CDC26 |
| 103 | CDC27 |
| 104 | CDKN1B |
| 105 | CDKN2B |
| 106 | CDKN2C |
| 107 | CDKN2D |
| 108 | CEBPB |
| 109 | CXCL8 |
| 110 | E2F1 |
| 111 | E2F2 |
| 112 | E2F3 |
| 113 | EED |
| 114 | EHMT1 |
| 115 | EHMT2 |
| 116 | EP400 |
| 117 | ERF |
| 118 | ETS1 |
| 119 | ETS2 |
| 120 | EZH2 |
| 121 | FOS |
| 122 | FZR1 |
| 123 | H1-0 |
| 124 | H1-1 |
| 125 | H1-2 |
| 126 | H1-3 |
| 127 | H1-4 |
| 128 | H1-5 |
| 129 | H2AB1 |
| 130 | H2AC14 |
| 131 | H2AC20 |
| 132 | H2AC4 |
| 133 | H2AC6 |
| 134 | H2AC7 |
| 135 | H2AC8 |
| 136 | H2AJ |
| 137 | H2AX |
| 138 | H2AZ1 |
| 139 | H2AZ2 |
| 140 | H2BC1 |
| 141 | H2BC10 |
| 142 | H2BC11 |
| 143 | H2BC12 |
| 144 | H2BC13 |
| 145 | H2BC14 |
| 146 | H2BC15 |
| 147 | H2BC17 |
| 148 | H2BC21 |
| 149 | H2BC3 |
| 150 | H2BC4 |
| 151 | H2BC5 |
| 152 | H2BC6 |
| 153 | H2BC7 |
| 154 | H2BC8 |
| 155 | H2BC9 |
| 156 | H2BS1 |
| 157 | H2BU1 |
| 158 | H3-3A |
| 159 | H3-3B |
| 160 | H3-4 |
| 161 | H3C1 |
| 162 | H3C10 |
| 163 | H3C11 |
| 164 | H3C12 |
| 165 | H3C13 |
| 166 | H3C14 |
| 167 | H3C15 |
| 168 | H3C2 |
| 169 | H3C3 |
| 170 | H3C4 |
| 171 | H3C6 |
| 172 | H3C7 |
| 173 | H3C8 |
| 174 | H4-16 |
| 175 | H4C1 |
| 176 | H4C11 |
| 177 | H4C12 |
| 178 | H4C13 |
| 179 | H4C14 |
| 180 | H4C15 |
| 181 | H4C2 |
| 182 | H4C3 |
| 183 | H4C4 |
| 184 | H4C5 |
| 185 | H4C6 |
| 186 | H4C8 |
| 187 | H4C9 |
| 188 | HIRA |
| 189 | HMGA1 |
| 190 | HMGA2 |
| 191 | ID1 |
| 192 | IFNB1 |
| 193 | IGFBP7 |
| 194 | IL1A |
| 195 | IL6 |
| 196 | JUN |
| 197 | KAT5 |
| 198 | KDM6B |
| 199 | LMNB1 |
| 200 | MAP2K3 |
| 201 | MAP2K4 |
| 202 | MAP2K6 |
| 203 | MAP2K7 |
| 204 | MAP3K5 |
| 205 | MAP4K4 |
| 206 | MAPK1 |
| 207 | MAPK10 |
| 208 | MAPK11 |
| 209 | MAPK14 |
| 210 | MAPK3 |
| 211 | MAPK7 |
| 212 | MAPK8 |
| 213 | MAPK9 |
| 214 | MAPKAPK2 |
| 215 | MAPKAPK3 |
| 216 | MAPKAPK5 |
| 217 | MINK1 |
| 218 | MIR24-1 |
| 219 | MIR24-2 |
| 220 | MOV10 |
| 221 | MRE11 |
| 222 | NBN |
| 223 | NFKB1 |
| 224 | PHC1 |
| 225 | PHC2 |
| 226 | PHC3 |
| 227 | POT1 |
| 228 | RAD50 |
| 229 | RB1 |
| 230 | RBBP4 |
| 231 | RBBP7 |
| 232 | RELA |
| 233 | RING1 |
| 234 | RNF2 |
| 235 | RPS27A |
| 236 | RPS6KA1 |
| 237 | RPS6KA2 |
| 238 | RPS6KA3 |
| 239 | SCMH1 |
| 240 | SP1 |
| 241 | STAT3 |
| 242 | SUZ12 |
| 243 | TERF1 |
| 244 | TERF2 |
| 245 | TERF2IP |
| 246 | TFDP1 |
| 247 | TFDP2 |
| 248 | TINF2 |
| 249 | TNIK |
| 250 | TNRC6A |
| 251 | TNRC6B |
| 252 | TNRC6C |
| 253 | TXN |
| 254 | UBA52 |
| 255 | UBB |
| 256 | UBC |
| 257 | UBE2C |
| 258 | UBE2D1 |
| 259 | UBE2E1 |
| 260 | UBE2S |
| 261 | UBN1 |
| 262 | VENTX |
