# Supplementary material for: Comprehensive genomics analysis of aging related gene signature to predict the prognosis and drug resistance of colon adenocarcinoma
Source: Front Pharmacol. 2023 Feb 28;14:1121634. doi: 10.3389/fphar.2023.1121634 (PMC10011090; doi:10.3389/fphar.2023.1121634)
Supplement: Supplementary file 6 [file DataSheet1.docx]

**Description of supplementary materials**

**Supplementary Fig 1** Dimensionality reduction of single cell. A, The cell number of 13 samples before and after filtering. B, TSNE diagrams overlapping of 13 samples. C, PCA diagrams of 13 samples. D, Anchor point after dimensionality reduction using PCA.

**Supplementary Fig 2** TSNE diagram of marker gene expression.

**Supplementary Fig 3** Comparisons of senescence-related pathway scores between risk groups. ns represents *P* > 0.05; **P* < 0.05, ***P* <0.01, ****P* <0.001, and *****P* <0.0001.

**Supplementary Fig 4** The methylation level for 11 risk genes in TCGA.

**Supplementary Table 1** A total of 262 genes obtained from four senescence-related pathways.
